# Reduced Processivity in a Chitobiohydrolase Enhances LPMO-Assisted Chitin Depolymerization

**DOI:** 10.1021/acs.biochem.5c00704

**Published:** 2026-02-17

**Authors:** Amanda K. Votvik, Zarah Forsberg, Alfonso Gautieri, Vincent G. H. Eijsink, Morten Sørlie

**Affiliations:** † Faculty of Chemistry, Biotechnology, and Food Science, The Norwegian University of Life Sciences (NMBU), 1432 Ås, Norway; ‡ Biomolecular Engineering Lab, Dipartimento di Elettronica, Informazione e Bioingegneria, Politecnico di Milano, 20133 Milano, Italy

## Abstract

Efficient enzymatic depolymerization of recalcitrant polysaccharides such as chitin and cellulose relies on processive glycoside hydrolases (GHs), whose efficiency can be enhanced through cooperation with lytic polysaccharide monooxygenases (LPMOs). For processive GHs, retained binding during successive catalytic events aids depolymerization of crystalline substrates but comes at the expense of slow dissociation (low *k*_off_) that limits turnover. Here, we engineered a series of mutants of the processive exochitobiohydrolase *Sm*ChiB from *Serratia marcescens*, in which a major determinant of substrate affinity and processivity, Trp220, was replaced by Tyr, Phe, His, Gln, or Ala. Functional analysis showed that these mutants had stepwise reductions in substrate affinity and processivity, with the latter being a signature of an increased *k*_off_. Molecular dynamics simulations confirmed that Trp220 plays an important role in substrate binding. The less processive *Sm*ChiB variants, and in particular W220Y, were able to reach wild-type-like performance only at high substrate concentrations. Importantly, the use of LPMOs to decrystallize the substrate and thereby increase its effective concentration, enhanced the performance of the less processive mutants to a much greater extent than for wild-type *Sm*ChiB. In some reaction setups, the combination of W220Y with an LPMO yielded twice as much soluble product compared to the wild-type enzyme under identical conditions. Thus, when combined with LPMOs, less processive GHs become more favorable because they are intrinsically more efficient catalysts when acting on noncrystalline substrates. These findings shed light on how the interplay between GHs and LPMOs can be optimized for efficient enzymatic conversion of recalcitrant polysaccharides.

## Graphical abstract

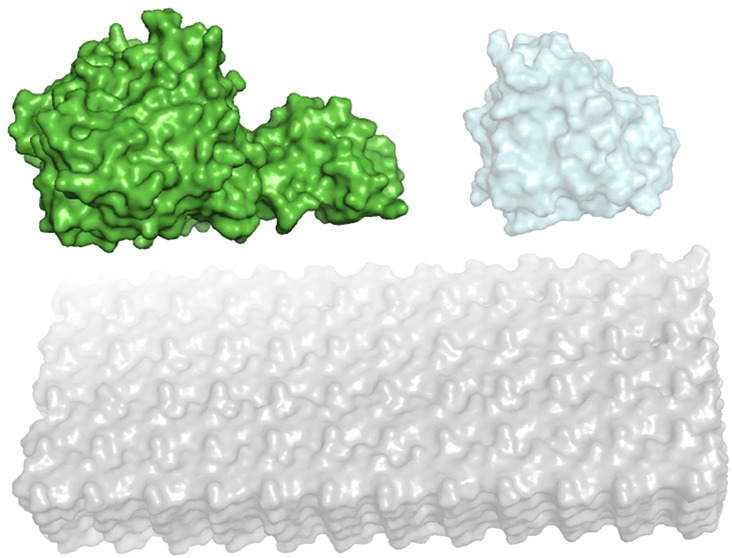

## Introduction

Chitin, a linear, insoluble polymer of β-1.4-linked *N*-acetylglucosamine, is the second most abundant biopolymer in the biosphere as well as a valuable renewable resource. However, the recalcitrance of chitin fibers presents a significant challenge in the industrial conversion of chitinous biomass. In Nature, aerobic depolymerization of recalcitrant polysaccharides like cellulose and chitin relies on the concerted action of endo- and exoacting glycoside hydrolases (GHs) and lytic polysaccharide monooxygenases (LPMOs).^1^ Processive GHs, i.e., exoacting GHs that remain attached to the polysaccharide chain between successive catalytic events, are of particular importance for the degradation of these insoluble, crystalline polysaccharides.^2–4^ One example is Chitinase B from *Serratia marcescens* (*Sm*ChiB), a thoroughly studied family 18 glycoside hydrolase (GH18) that acts processively from the nonreducing end of chitin polymers, releasing *N*,*N*′-diacetylchitobiose through a substrate-assisted retaining mechanism where the *N*-acetyl group of the sugar unit in subsite −1 acts as the nucleophile.^5–8^

During processive action, GHs slide along the polysaccharide chain catalyzing multiple cuts that generate dimeric products.^9–11^ Maximizing product formation per binding event is especially advantageous for enzymes targeting insoluble polysaccharides, where accessible binding sites are limited, enzyme rebinding is energetically costly, and free chain ends are prone to reassociation with the crystal lattice.^2,6,12^ There is, however, a trade-off between processivity and catalytic efficiency, since the low off-rates required for processivity inherently limit catalytic turnover.^6,13^ High substrate affinity may also increase the formation of nonproductive substrate complexes.^14,15^ Still, maintaining high substrate affinity is crucial, given the clear correlation between processivity and substrate binding free energy.^16^ The fine balance between binding and turnover that is required for processive hydrolysis of insoluble substrates reflects the Sabatier principle: optimal catalytic efficiency is achieved at intermediate binding affinities.^15,17^

To degrade crystalline chitin, *S. marcescens* employs an enzyme cocktail consisting of two processive exochitinases, ChiA and ChiB, an endochitinase, ChiC, an *N*-acetyl glucosaminidase, and an LPMO called *Sm*AA10A or CBP21 (name used from now on).^3,18,19^ While the active sites of chitinases form clefts or tunnels that catalyze hydrolysis, LPMOs feature a flat substrate-binding surface that enables interaction with crystalline regions, where chain cleavage occurs through catalytic oxidation.^3,10,20^ The active site of LPMOs consists of two histidine residues coordinating a copper cofactor, often referred to as the histidine brace.^21^ Upon reduction by an electron donor, the copper center reacts with O_2_ or H_2_O_2_ to generate a highly reactive oxygen species that abstracts a hydrogen atom from the C1 or the C4 carbon in the scissile glycosidic bond, leading to hydroxylation and bond breakage.^22^ Of note, only C1-oxidation has been detected for chitin-active LPMOs. Originally, LPMOs were thought to be monooxygenases, using O_2_, whereas recent data suggest that they are peroxygenases, using H_2_O_2_.^23^ Importantly, in typical reductant-driven LPMO reactions, H_2_O_2_ is generated *in situ* through the oxidase activity of the enzyme and abiotic oxidation of the reductant.^24–26^ LPMO activity is thought to disrupt the surface of crystalline regions in the substrate and create new access points for processive GHs.^27,28^

The interplay between processive GHs and LPMOs has been explored in numerous studies, mainly focusing on how LPMOs boost the activity of GHs and, thus, the overall efficiency of substrate degradation.^29^ One key remaining question is to what extent the action of LPMOs can reduce the reliance on intrinsically slow and “sticky” processive GHs. Studies of cellulases and chitinases have shown that the processivity and “stickiness” of these GHs is modulated by aromatic residues lining the substrate-binding clefts. As an example, in *Sm*ChiB, residues Trp97, Phe190, and Trp220 form an aromatic clamp interacting with sugars bound in the +1, +2, and +3 subsites (Figure 1) and mutation of these residues leads to reduced processivity and reduced enzyme efficiency in the degradation of crystalline substrates.^6,30^

**Figure 1:**
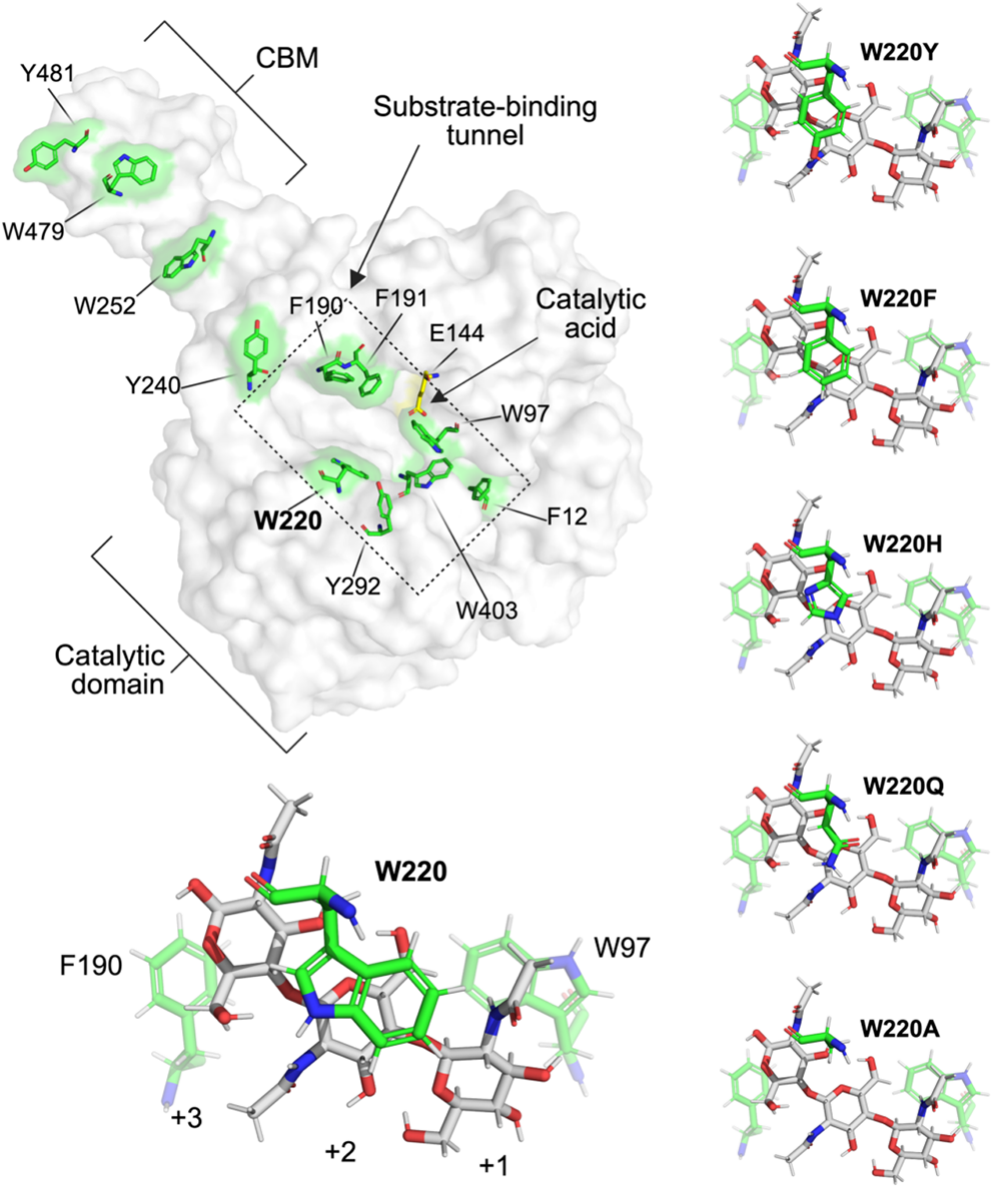
Overall structure of *Sm*ChiB and substrate binding at subsites +1 to +3. The top left panel displays the substrate-binding surface of wild-type (WT) ChiB, highlighting aromatic residues thought or known to be involved in substrate binding (green), and the catalytic acid Glu144 (yellow). The catalytic domain and carbohydrate-binding module (CBM) are labeled, along with the substrate-binding tunnel and catalytic site. Trp220 (W220) is highlighted in bold. The bottom left panel shows substrate-binding subsites +1, +2, and +3 (aromatic clamp) of the wild-type enzyme bound to chitotriose (pdb, 1E6N).^7^ The right panels depict the modified aromatic clamps of the variants bound to chitotriose, in the initial configuration.

In an early study, Hamre et al. showed that the detrimental effects of processivity-reducing mutations on the activity of GHs on crystalline chitin can be counteracted by supplying the reaction with an LPMO.^31^ One variant of *Sm*ChiB, *Sm*ChiB-W220A, stood out, because, when combined with the same LPMO, this variant appeared to be more efficient than the wild-type enzyme. This interesting observation suggests that it may indeed be possible to optimize chitin degradation by carefully tuning GH processivity and using LPMOs. To further assess the potential of this strategy, we have generated and functionally characterized a series of *Sm*ChiB variants with different residues at position 220 and, thus, different substrate affinities. One underlying hypothesis was that moderate reduction of substrate interactions mediated by residue 220, rather than eliminating these entirely through the W220A mutation, could optimize the interplay between less processive *Sm*ChiB variants and LPMOs. The results show that the nature of this key residue plays a major role in the processive ability of *Sm*ChiB and strongly affects its interplay with LPMOs.

## Materials and Methods

### Molecular Dynamics Simulations

The initial protein–substrate configuration was derived from the crystal structure of *Sm*ChiB bound to (GlcNAc)_5_ (PDB: 1E6N),^7^ excluding the sugar units occupying the −1 and −2 subsites to destabilize the complex. Mutant structures (W220A, W220H, W220Q, W220Y, W220F) were generated using the Mutagenesis Wizard in PyMOL (version 3.0.3) (Figure 1). All six variants, including the wild type, were simulated in triplicates. The models were solvated with a 10 Å layer of TIP3P water and Na^+^/Cl^–^ ions to neutralize the system charge, resulting in a final simulation box of ≈60.000 atoms. The models were subjected to 5000 steps of energy minimization with 5 kcal·mol^–1^·Å^–2^ positional restraints on the backbone atoms of the enzyme and on the chitotriose. The systems were then heated linearly from 0 to 300 K for 100 ps at a constant volume, with restraints lowered to 1 kcal·mol^–1^·Å^–2^, using the Langevin thermostat with a collision frequency of 1 ps^–1^. Density equilibrations were performed at 300 K for 1 ns at a constant pressure of 1 atm using the Berendsen barostat with a pressure relaxation time of 1 ps. The final 50 ns equilibration step was performed in the NVT ensemble. The 100 ns production runs were performed using the same conditions as in the final equilibration step, except that all positional restraints were removed. In all simulations, we used a time step of 2 fs, periodic boundary conditions with a 12 Å cutoff for nonbonded interactions, and PME treatment of long-range electrostatics. Simulations were performed using the CUDA version of NAMD3 and the CHARMM 36 force field.^32^ Substrate retention during the unrestrained MD simulations was assessed by monitoring the RMSD of chitotriose relative to the starting structure. Preprocessing and postprocessing of the MD simulations were performed using VMD and PyMOL.^33^

### Materials

Chemicals were obtained from Sigma-Aldrich (St. Louis, Missouri, USA) unless stated otherwise. All stocks and reactions were prepared using double-distilled water (ddH_2_O) produced by a Milli-Q Direct 16 system (Merck, Darmstadt, Germany). β-chitin from squid pen (GC2112; Glentham Life Sciences Ltd., Corseham, UK) was milled in-house using zirconium oxide beads in a PM 200 planetary ball mill (Retsch, Haan, Germany) and sieved to obtain particles smaller than 75 μm. β-chitin stocks were hydrated for at least 24 h (4 °C) prior to use. Ascorbic acid was prepared as a 200 mM stock solution, aliquoted for single use, and stored at −20 °C. Noninducing media (MDG) and autoinducing media (ZYP-5052) were prepared according to Studier (recipes available at [link]),^34^ except that the 1000× trace element solution was replaced with a minimal 50 mM FeCl_3_ solution. All solutions, except β-chitin suspensions, were sterilized after preparation by autoclaving at 121 °C for 20 min or filtration through Millipore Steritop vacuum-filters with a 0.22 μm membrane (Merck, Darmstadt, Germany).

### Expression and Purification of Recombinant Chitinases

Wild-type *Sm*ChiB originates from *Serratia marcescens* strain BJL200.^5^ Genes encoding wild type *Sm*ChiB and its mutants, fused to an N-terminal PelB signal peptide, were ordered codon optimized from GeneScript in the expression vector pET-26b­(+). Expression vectors were transformed into One Shot BL21 Star (DE3) chemically competent *Escherichia coli* cells (Invitrogen), according to the supplier’s protocol.

Colonies from freshly transformed expression strains were grown to saturation in 2.5 mL of noninducing medium supplemented with 100 μg mg^–1^ kanamycin at 37 °C and shaking at 200 rpm. A 0.5 mL aliquot of this culture was then used to inoculate 0.5 L of autoinducing medium supplemented with 400 μg mg^–1^ kanamycin, which was incubated at 37 °C with shaking at 200 rpm for 2 h before the temperature was reduced to 20 °C and the culture was further incubated for 3 days. Cells were harvested by centrifugation, and periplasmic proteins were extracted using cold osmotic shock with magnesium, as described previously.^35^ After extraction, the periplasmic fraction was sterilized by filtration through a 0.22 μm syringe filter (Merck, Darmstadt, Germany).

Chitinases were purified from periplasmic extracts with hydrophobic interaction chromatography. Periplasmic extracts were adjusted to 20 mM Tris-HCl (pH 8.0) and 0.4 M (NH_4_)_2_SO_4_ (buffer A) and thereafter loaded onto two tandem coupled HiTrap PhenyL FF columns (5 mL) (GE Healthcare) connected to an ÄKTA purifier FPLC system (GE Healthcare). After complete loading at a flow of 1 mL min^–1^, the unbound protein was washed out using 20 mM Tris-HCl, pH 8.0 (buffer B) at a flow of 2 mL min^–1^, before elution with 20 mM Tris-HCl (pH 8.0) and 20% isopropanol (buffer C) was achieved through a 0–100% gradient over 10 min. In contrast to wild-type ChiB, all the mutant variants started to elute while washing with buffer B, and the gradient with buffer C was used to speed up the elution. Of note, most native *E. coli* proteins eluted during the loading of the periplasmic extracts or during washing step with buffer B.

Fractions containing protein of high purity, analyzed by SDS-PAGE, were pooled, concentrated, and buffer-exchanged to 20 mM Tris-HCl (pH 8.0) using Amicon Ultra-15 centrifugal filters with a 30 kDa molecular weight cutoff (Merck, Darmstadt, Germany), before storing at 4 °C. Final enzyme concentrations were estimated from total protein concentrations determined by the Bradford assay, corrected for enzyme purity as assessed by SDS-PAGE.

*Sm*AA10A (CBP21), *Sc*AA10D and *Af*AA11A were produced, purified and copper saturated as described previously.^36,37^

### Chitin Degradation by Wild-Type *Sm*ChiB and Mutant Variants with and without LPMO

All reactions were performed in triplicates carried out in 2.0 mL microtubes using an Eppendorf ThermoMixer Comfort (Eppendorf, Hamburg, Germany) set to 37 °C with shaking at 800 rpm. Reaction mixtures contained 100 nM Chitinase, 20 mg mL^–1^ β-chitin (unless stated otherwise), and 50 mM phosphate buffer, pH 6.0. Reactions lacking LPMO (LPMO-free setup) were supplemented with 1 mg mL^–1^ BSA (bovine serum albumin) to minimize nonspecific enzyme adsorption to tube surfaces. For reactions with LPMO (LPMO-containing setup), varying LPMO concentrations were preincubated with substrate and buffer for 30 min before initiating LPMO activity by adding ascorbic acid as the reductant, or an equal volume of ddH_2_O for control reactions without LPMO activity. After an additional 30 min or 2.5 h incubation with activated or resting state LPMO, Chitinase was added to start the time course. Enzyme-free control reactions were included in all experiments: buffer and substrate were incubated with or without BSA (LPMO-free setup), or, with or without ascorbic acid (LPMO-containing setup).

For both setups, aliquots were collected at defined time points and mixed 1:1 with 20 mM H_2_SO_4_ to terminate enzymatic activity. Samples were thereafter filtered through a MultiScreen 96-well filter plate (Merck, Darmstadt, Germany) to remove denatured protein and residual chitin particles, then stored at 4 °C (analyzed within 24 h) or −20 °C until HPLC analysis.

### End Point Analysis of Hydrolytic Activity across Varying Substrate Concentrations

Chitin degradation was performed and sampled as described for the LPMO-free setup, using β-chitin concentrations of 2, 10, 20, and 30 mg mL^–1^. Samples were collected 2 h after starting the reactions by Chitinase addition.

### Time Resolved Degradation by *Sm*ChiB Variants in the Presence of CBP21

Reaction mixtures contained 1 μM CBP21 either activated with 2 mM ascorbic acid or kept inactive by adding ddH_2_O (controls without LPMO activity). The mixtures were further incubated for 2.5 h prior to Chitinase addition. Samples were collected every hour for up to 6 h. All other reaction and sampling conditions were as described for the LPMO-containing setup.

### Extended Chitinase Incubation with CBP21, *Sc*AA10D, and *Af*AA11A

Reaction mixtures containing 1 μM LPMO were activated with 1 mM ascorbic acid (or ddH_2_O: controls without LPMO activity) and incubated for 30 min before Chitinase addition. β-chitin was supplemented with sodium azide (final concentration 0.01%) to prevent microbial growth in reactions. Samples were taken after 1, 3, 6, 13, 24, and 48 h. Otherwise, all reaction and sampling conditions were as described for the LPMO-containing setup.

### LPMO-Dosage Response with CBP21

Reaction mixtures containing either 0, 0.1, 0.5, 1, 2, 5, or 10 μM CBP21 were activated with 1 mM ascorbic acid (or ddH_2_O: controls without LPMO activity) and incubated for 30 min prior to Chitinase addition. Samples were collected every hour for up to 5 h. All other reaction and sampling conditions were as described for the LPMO-containing setup.

### Quantification of Chitin-Derived Products

Chromatographic analysis of native and oxidized products was performed using a 100 mm × 7.8 mm Rezex RFQ-Fast Acid H+ (8%) column (Phenomenex, Torrance, CA, USA) operated at 85 °C by a Dionex Ultimate 3000 UHPLC system (Dionex Corp., Sunnyvale, CA, USA), with 5 mM H_2_SO_4_ as the mobile phase. Sample sizes were 4–8 μL and chitin derived products were eluted isocratically at 1 mL min^–1^, with a total run time of 6 min. Analytes were monitored by measuring absorbance at 194 nm.

Quantification was performed by creating individual standard curves for the native monomer (GlcNAc), dimer and trimer, and oxidized dimer (GlcNAcGlcNAc1A), trimer and tetramer. The oxidized standards were created in-house by incubating individual reactions of native dimer, trimer and tetramer (Megazyme, Bray, Ireland, 95% purity) with a chitooligosaccharide oxidase from *Fusarium graminearium*, as described previously.^38^

## Results and Discussion

### Computational Analysis of SmChiB Variants with Varying Substrate Affinity

We generated four additional variants of *Sm*ChiB that, together with the W220A mutant, provide a series with progressively weakened favorable contacts with the substrate. Mutants W220Y and W220F both retain aromaticity, while the indole is replaced by a less bulky benzene. The two mutants differ in terms of polarity and the potential of hydrogen-bonding through the side chain. W220H places an aromatic heterocycle (imidazole) at position 220 with inherent polarity. W220Q offers polarity without aromaticity, while W220A is thought to eliminate substrate interactions altogether.

Substrate affinity was assessed by molecular dynamics (MD) simulations of wild-type *Sm*ChiB and mutated variants in complex with (GlcNAc)_3_ (trimer) bound to subsites +1 to +3. The initial protein–substrate configuration was derived from the crystal structure of *Sm*ChiB bound to (GlcNAc)_5_ (PDB: 1E6N, chain A),^7^ excluding the sugar units occupying the −1 and −2 subsites. Mutant structures were generated using the Mutagenesis Wizard in PyMOL (version 3.0.3). All six variants, including the wild type, were simulated in triplicates. Systems were equilibrated for 50 ns with positional restraints on both the substrate and the protein backbone, followed by unrestrained 100 ns production runs. Interatomic contacts in equilibrated protein–substrate models were computed with Arpeggio and visually inspected in PyMOL to assess putative substrate interactions.^39^ The equilibrated models are shown in the lower and right panels of Figure 1. Substrate retention (*n* = 3 productive runs) during the subsequent unrestrained simulations was assessed by monitoring the RMSD of chitotriose relative to the starting structure (Figure S1). See Web Enhanced Objects 1–6 for representative unrestrained trajectories.

Throughout the unrestrained MD simulation of wild-type *Sm*ChiB, the trimer remained stably bound, consistent with stabilizing CH−π contacts with Trp220, in addition to Trp97 and Phe190. For W220Y and W220F, the most noticeable differences relative to the wild-type enzyme were increased side-chain rotational freedom of residue 220 and increased conformational freedom of the nearby side chain of Arg294 that caused it to lose its interaction with the *N*-acetyl carbonyl of the nonreducing-end GlcNAc unit. Consequently, the trimer showed greater mobility within the cleft but remained engaged through contacts with Trp97 and Phe190, alongside intermittent interactions with the substituted residue. In the W220H and W220Q mutants, residue 220 exhibited high side-chain flexibility and even weaker substrate contacts, and for these mutants, substrate displacement from the binding cleft was observed (Figure S1). In W220A, interactions between residue 220 and the trimer were not observed. In this mutant, the trimer displayed a high mobility within the cleft, while being loosely retained through interactions with Trp97. It is worth noting that W220A seems to retain the substrate slightly better than the W220H and W220Q mutants (Figure S1).

The results suggest that substrate binding decreases as the aromatic surface of residue 220 is reduced. This is in line with the well-recognized importance of CH−π interactions in carbohydrate binding.^40^ The strength of such CH−π contacts tends to scale with the π-electrostatic potential and polarizability of the side chains (Trp > Tyr > Phe > His).^41^ As for Tyr versus Phe, phenylalanine is purely apolar, whereas the neutral phenolic −OH of tyrosine donates electron density into the aromatic ring, rendering its π-face more negative.^41^ The imidazole of histidine provides a smaller than Trp/Tyr/Phe, while offering hydrogen bonding. Based on PROPKA 3,^42^ the introduced histidine in the W220H mutant is predicted to have a p*K*_a_ of 5.79, corresponding to 38% protonation at pH 6.0.

### Mutational Effects on Hydrolytic Activity

During degradation of insoluble chitin, *Sm*ChiB primarily produces chitobiose (dimer) through initial cleavage and subsequent processive action, as well as chitotriose (trimer) that may result from the first cleavage after a productive binding event.^11^ The trimer serves as a soluble substrate that is gradually converted into dimers and *N*-acetylglucosamine (monomer). Monomer production thus reflects initial binding events, whereas the total dimer yield includes products derived from the initial cleavage, processive action, and secondary conversion of soluble trimers. Consequently, the total depolymerization of β-chitin can be represented by the combined levels of monomers, dimers, and residual trimers after converting all products to monomer equivalents (GlcNAc eq.). To determine the degree of substrate solubilization, monomer equivalents were expressed as a percentage of the theoretical maximum monomer yield. Figure 2 shows that the wild type, presumed to have the highest substrate affinity, indeed exhibited the highest degradation efficiency at the lowest tested β-chitin concentration.

**Figure 2:**
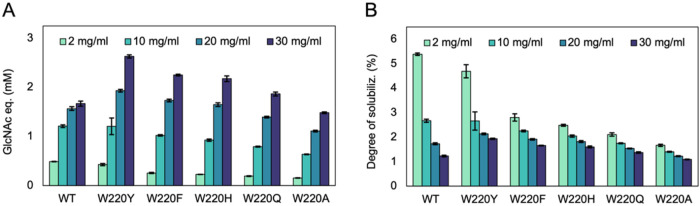
End point analysis of hydrolytic activity on solid β-chitin across varying substrate concentrations. Enzymatic degradation was assessed after 2 h of incubation with increasing concentrations of β-chitin (2, 10, 20, and 30 mg mL^–1^). (A) Total yield of soluble products expressed as monomer equivalents (GlcNAc eq.). (B) Degree of solubilization (see text for further explanation). All reactions contained 100 nM Chitinase, 0.1 mg mL^–1^ BSA in 50 mM sodium phosphate buffer (pH 6.0) and were incubated at 37 °C with shaking at 800 rpm. Reactions were initiated by addition of enzyme and terminated by diluting 1:1 with 20 mM H_2_SO_4_. Controls including Chitinase-free reactions, with and without BSA, showed no increase in product levels over time (data not shown). Error bars represent standard deviations for three independent reactions.

The data for the wild type also display a substrate concentration-dependent increase in activity that approaches a plateau at the highest substrate concentration tested (Figure 2A), consistent with near-saturation kinetics. Interestingly, although all mutants showed lower activity than the wild type at the lowest substrate concentration, several variants outperformed the wild type at higher substrate concentrations, W220Y being the most efficient, followed by W220F, W220H, and W220Q, which displayed progressively lower product levels. The W220A mutant consistently exhibited the lowest performance and failed to exceed the wild type at any substrate concentration tested. Notably, careful inspection of Figure 2A reveals that for the W220Y, W220F, W220H, and W220Q variants, the ability to outperform the wild type becomes increasingly dependent on the substrate concentration. As an example, W220Q reached wild-type-like product levels only at the highest substrate concentration. None of the variants showed the near-saturation kinetics observed for the wild type.

These results show that at low substrate concentrations, where the degradation reaction may be association limited, the substrate affinity provided by Trp220 is an important determinant of enzyme efficiency, consistent with a previous study on *Sm*ChiB-W220A that employed lower substrate concentrations and accordingly reported a larger mutational effect.^6^ On the other hand, at higher substrate concentrations, mutations that reduce the substrate affinity mediated by residue 220 are beneficial for activity, suggesting that the limiting step of the reaction shifts toward the rate of dissociation. A positive correlation between lower substrate affinity and higher catalytic turnover has also been observed for both engineered and wild type processive cellulases, where the optimal substrate affinity depends on the applied substrate concentration.^15,43^

### Comparative Analysis of Apparent Processivity on β-Chitin

Considering processivity is crucial for understanding the mechanism of chitin hydrolysis by chitobiohydrolases. However, due to the biphasic nature of the experimental system, it is not straightforward to measure and quantify the degree of processivity. A pragmatic approach is to determine apparent processivity (*P*_app_) by the division of dimer minus monomer concentrations with the sum of trimer and monomer concentrations according to (eq 1)^11,44^

1 $$P_{\mathrm{app}}=\left(\left[\mathrm{dimers}\right]-\left[\mathrm{monomers}\right]\right)/\left(\left[\mathrm{trimers}\right]+\left[\mathrm{monomers}\right]\right)$$

Despite certain limitations,^11^ this approach enables a qualitative assessment of differences in processivity between the wild type and the mutants. Processivity was assessed for 2 h reactions with varying substrate concentrations (2–30 mg mL^–1^; Figure 3A) and, in addition, over a 6 h time course in a reaction with 20 mg mL^–1^ substrate (Figure 3B).

**Figure 3:**
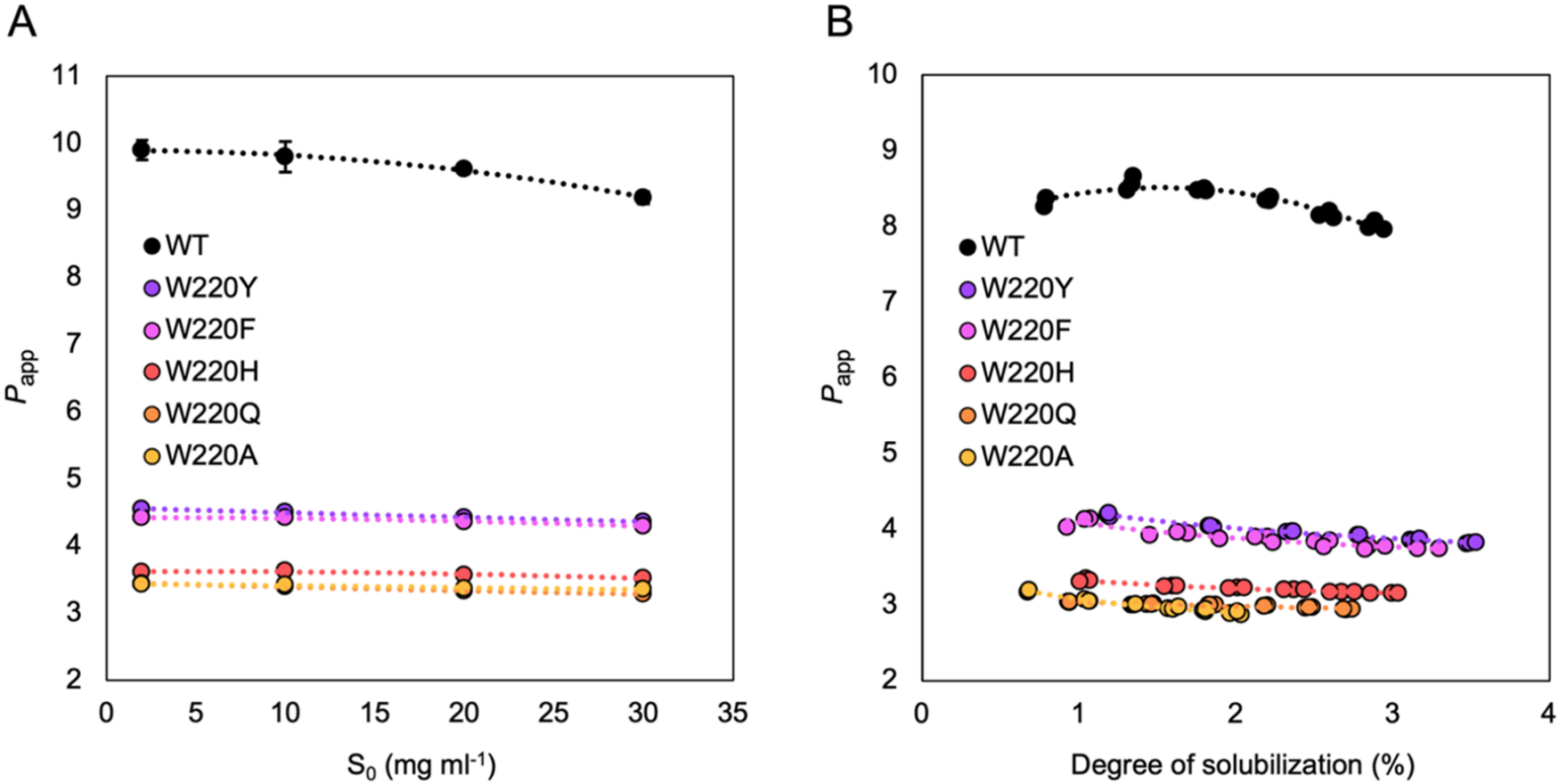
Apparent processivity (*P*_app_) as a function of substrate concentration or degree of solubilization. (A) End point *P*_app_ after the 2 h incubation shown in Figure 2 with increasing amounts of β-chitin (2–30 mg mL^–1^). (B) *P*_app_ for the reductant-free reactions shown in Figure 4 with 20 mg mL^–1^ β-chitin plotted against the degree of solubilization over the 6 h time course. Error bars in panel (A) denote standard deviations (*n* = 3), while all individual data points are plotted in panel (B). Dotted lines in both panels are guides for the eye (no model fitting).

The results show that substitution of Trp220 led to a substantial reduction in *P*_app_ that was largely independent of the substrate concentration (Figure 3A) and the extent of substrate degradation (Figure 3B). Clearly, the tryptophane at position 220 is important for processivity, as all variants showed a considerably reduced *P*_app_. In terms of *P*_app_, a coarse ranking of the *Sm*ChiB variants is WT > W220Y ≈ W220F > W220H > W220Q ≈ W220A. The two mutants containing noncharged aromatic residues in place of Trp220, namely W220F and W220Y, show clearly higher processivity than the other mutants.

All in all, the computational and experimental results described so far show that mutation of Trp220 leads to enzymes with reduced substrate affinity and processivity, and to lower catalytic efficiency when the substrate concentration is low. In accordance with the notion of a trade-off between substrate affinity and processivity on the one hand, and the catalytic rate on the other, some mutants are in fact more active than the wild-type enzyme at high substrate concentrations, where the reaction is expected to shift from being association-limited to dissociation-limited.

Beckham and Crowley reported energies of 8.0 kcal mol^–1^ (interior) and 5.6 kcal mol^–1^ (edges) per chitobiose unit peeled from α-chitin.^45^ These energies, while potentially somewhat lower for β-chitin, illustrate the energetic barriers that processive chitinases must overcome. Interestingly, in previous studies, Hamre et al. and Jana et al. estimated the energetic effects of the F190A and W97A substitutions on binding of (GlcNAc)_6_ to be modest (ΔΔ*G*_bind_ ≈ +0.6 and +0.9 kcal mol^–1^, respectively), compared to a much larger effect of the W220A mutation (ΔΔ*G*_bind_ ≈ +3.4 kcal mol^–1^).^30,46^ These results show that Trp220 is the main contributor to favorable binding in the positive subsites, and that the binding energy provided by this residue approaches the values calculated by Beckham and Crowley.

### Chitin Degradation by *Sm*ChiB Variants in the Presence of an LPMO

The well-characterized CBP21 from *S. marcescens* was used to study the interplay between the *Sm*ChiB variants and a chitin-active LPMO. The β-chitin (20 mg mL^–1^) was pretreated with LPMO for 2.5 h in the presence of a reductant, followed by a 6 h incubation with Chitinase. Figure 4 displays the time-resolved solubilization of chitin, in reactions with and without LPMO activity, represented as the combined levels of generated dimers and monomers (in GlcNAc equivalents). Other products include chitotriose and, in reactions with LPMO activity, oxidized chitobiose, which could not be quantified because these two compounds showed similar retention times and thus could not be separated. Both these products are minor, and the error introduced by neglecting them is below, and in most cases well below, 10% (see the legend of Figure 4 for details). Oxidized trimers and tetramers were detected at similar levels across all reactions with active LPMO, whose abundances were much lower than those of the native dimers and monomers used for quantification (Figure S2).

**Figure 4:**
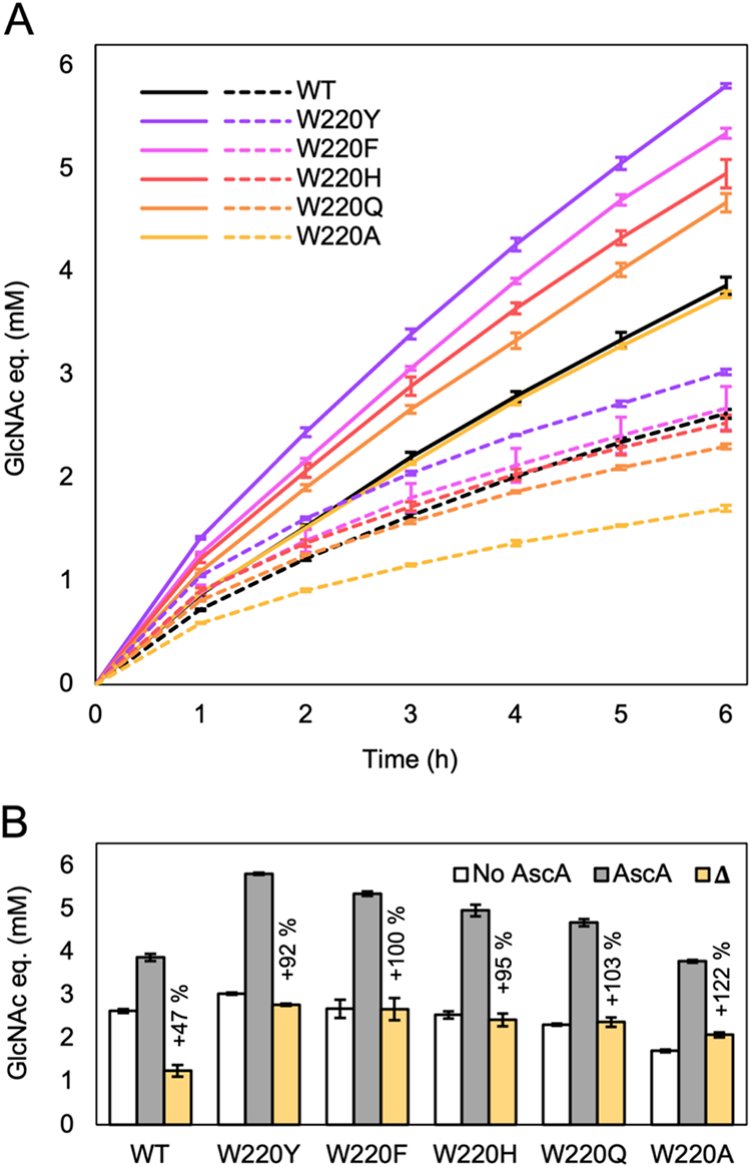
Chitin solubilization by *Sm*ChiB variants in the absence or presence of LPMO activity. All reactions contained both Chitinase and LPMO and were run either without (dashed lines) or with (solid lines) LPMO activity, depending on the addition of ascorbic acid (reducing agent). Chitin solubilization was quantified as the total amount of *N*-acetylglucosamine (GlcNAc) equivalents, based on the formation of GlcNAc and chitobiose. (A) Time course of chitin solubilization. (B) End point levels of chitin solubilization in reactions with ascorbic acid (active) and without nonactive, the difference between these two conditions (Δ), and the corresponding relative enhancement (%; Δ values as a percentage of the No ascorbic acid level). Minor amounts of both trimers (maximum GlcNAc eq ≤ 120 μM) and, in reactions with active LPMO, oxidized dimers (maximum GlcNAc eq ≤ 400 μM), were excluded from the displayed results due to similar retention times. Across *Sm*ChiB variants, LPMO-active reactions also displayed comparable fractions of oxidized chitotriose and oxidized chitotetraose, together representing about 300 μM GlcNAc (see Figure S2). All reactions contained 100 nM Chitinase, 1 μM LPMO (CBP21), and 20 mg mL^–1^ β-chitin in 50 mM phosphate buffer (pH 6.0) and were incubated at 37 °C with shaking at 800 rpm. The LPMO was preincubated with β-chitin for 30 min, followed by a second 2.5-h preincubation in the absence or presence of 2 mM ascorbic acid, before initiating the final 6-h incubation by adding the Chitinase. In reactions lacking LPMO activity, the reducing agent was replaced by an equal volume of dH_2_O. Samples were collected hourly starting at 1 h after the addition of the Chitinase. Enzyme-free control reactions, incubated with or without ascorbic acid, did not display any significant product formation (not shown). Error bars represent standard deviations from triplicate reactions.

The progress curves in Figure 4A and the end point analysis in Figure 4B show that LPMO activity, expectedly, enhanced product formation. Importantly, the gain in product formation caused by the LPMO is much more pronounced for several of the *Sm*ChiB variants (Figure 4B). In reactions with active LPMO, all mutants, except W220A showed a greater performance than the wild-type enzyme, with W220Y displaying the highest degree of solubilization, followed by W220F, W220H and W220Q. Notably, although the W220A variant did not reach wild-type activity when supplemented with an active LPMO, it displayed the highest relative enhancement (Figure 4B), underpinning the importance of LPMO activity when using a *Sm*ChiB variant with low processivity.

For the reactions without LPMO, it is interesting to note that the progress curve for the wild-type enzyme crosses several of the progress curves for the mutants, suggesting slower rate decay over time. The slightly increased rate decays observed for the variants align with weaker binding and faster dissociation, which becomes increasingly disadvantageous as the more recalcitrant fraction of the substrate increases, and the need for strong binding and processivity becomes more critical.

Figure 4A,B lead to the remarkable conclusion that, when LPMOs take part in the degradation reaction, chitin degradation becomes more efficient when using less processive variants of *Sm*ChiB. It has been shown in some experimental setups that the synergy between LPMOs and GHs is mutual.^47^ Indeed, one could envisage that the Chitinase, by peeling off chitin chains from a fiber surface amorphized through LPMO action, helps the LPMO gain access to new productive binding sites in the underlying crystalline material. In principle, therefore, the large differences in product levels observed in Figure 4 could also arise from differences in the extent to which the Chitinase boosts LPMO activity. However, all *Sm*ChiB variants yielded similar levels of LPMO-generated oxidized products (Figure S2).

The rate constant for *Sm*ChiB acting on soluble oligomers is on the order of 20 s^–1^,^48^ whereas the rate constant for degradation of β-chitin is approximately 1.0 s^–1^.^6^ The rate constant for CBP21 in an ascorbic acid–driven reaction is one to 2 orders of magnitude lower, estimated at approximately 0.02 s^–1^.^19^ Since the LPMO reaction itself is clearly rate-limiting, variation in the ability of the *Sm*ChiB variants to release oxidized fragments from the substrate and degrade LPMO-generated longer oxidized oligomers caused by mutating Trp220, will not affect the final levels of short oxidized (and nonoxidized) oligomers.

### Assessing Enhancement of Chitin Hydrolysis in Extended Reactions and with Other LPMOs

To assess whether the observed LPMO effects on chitin hydrolysis extended to other LPMOs and longer reaction times, we examined the interplay between selected *Sm*ChiB variants and three different LPMOs in 48h reactions. Three *Sm*ChiB variants were used, the wild type, the high-performing W220Y variant, and the low-performing W220A variant. In addition to CBP21, two single-domain LPMOs were tested: the catalytic domain of the two-domain *Sc*AA10D from *Streptomyces coelicolor* and *Af*AA11A from *Aspergillus fumigatus*, which is naturally a single-domain protein like CBP21.^36^ Compared to the reactions shown in Figure 4, the second preincubation step, involving LPMO with or without reductant, was shortened to 30 min, and the reductant concentration was halved to 1 mM to maintain a lower yet more stable level of LPMO activity throughout the extended reaction period.

For all three LPMOs, the increase in product formation caused by LPMO activity was higher for the two less processive Chitinase variants, compared to wild-type *Sm*ChiB (Figure 5).

**Figure 5:**
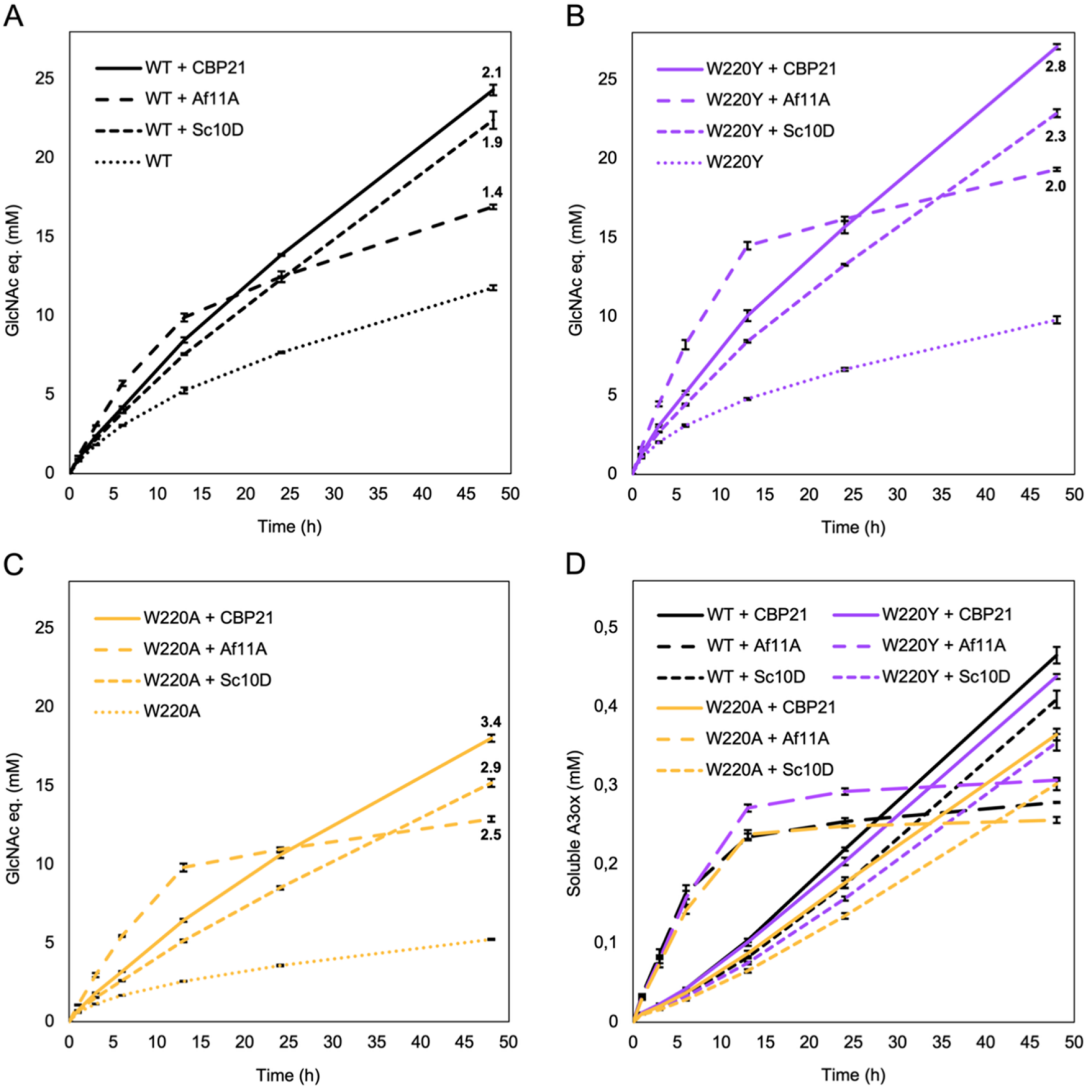
Progress curves for extended chitin degradation by *Sm*ChiB-WT, W220Y, and W220A in the absence or presence of LPMO activity provided by three different LPMOs. (A–C) Chitin solubilization, expressed as GlcNAc equivalents representing the combined level of dimers and monomers, is shown for reactions wild-type ChiB, W220Y, or W220A, respectively, in the absence or presence of active LPMO. The curves for the LPMO-containing reactions are labeled with the ratio between the final product level in the reaction and the product level obtained in the reaction without active LPMO. (D) Formation of oxidized trimers in the LPMO-reactions depicted in panels (A–C). All reactions contained 100 nM Chitinase, 1 μM LPMO, 20 mg mL^–1^ β-chitin, and 0.01% sodium azide in 50 mM phosphate buffer (pH 6.0) and were incubated at 37 °C with shaking at 800 rpm. The LPMO reaction was fueled with 1 mM ascorbic acid; in control reactions lacking LPMO activity, the reaction was supplemented with dH_2_O instead of reductant. Each LPMO was preincubated with substrate for 30 min, followed by a second 30 min preincubation, in the absence or presence of 1 mM ascorbic acid, before adding the Chitinase. Enzyme-free control reactions, incubated with or without ascorbic acid, did not display any significant product formation (not shown). Error bars represent standard deviations from triplicate reactions.

Furthermore, the hydrolysis-enhancing effect of the LPMO was larger for W220A than for W220Y, in all three cases. These results align well with the results shown in Figure 4B and confirm that less processive enzymes benefit more from LPMO activity than more processive enzymes. The different LPMOs yielded different progress curves, with *Af*AA11A showing a clear difference relative to CBP21 and *Sc*AA10D. Figure 5D reveals a possible underlying cause: *Af*AA11A was markedly more active in the initial phase of the reaction, whereas formation of oxidized products ceased after approximately 12 h; in contrast, CBP21 and *Sc*AA10D showed slower but steady product formation over the complete reaction time. These differences reflect functional differences between LPMOs related to, for example, their substrate affinity and their (H_2_O_2_-generating) oxidase activity. Existing data indicate that AA11-type LPMOs have much higher oxidase activity than AA10 LPMOs;^49^ high production of H_2_O_2_ would lead to high initial LPMO activity and relatively rapid cessation of the reaction due to reductant depletion and/or enzyme inactivation, which is exactly what the progress curves in Figure 5D show. Of note, Figure 6D also shows that LPMO action was not affected by the nature of the *Sm*ChiB variant, in accordance with data for the 6 h reactions depicted in Figure S2.

**Figure 6:**
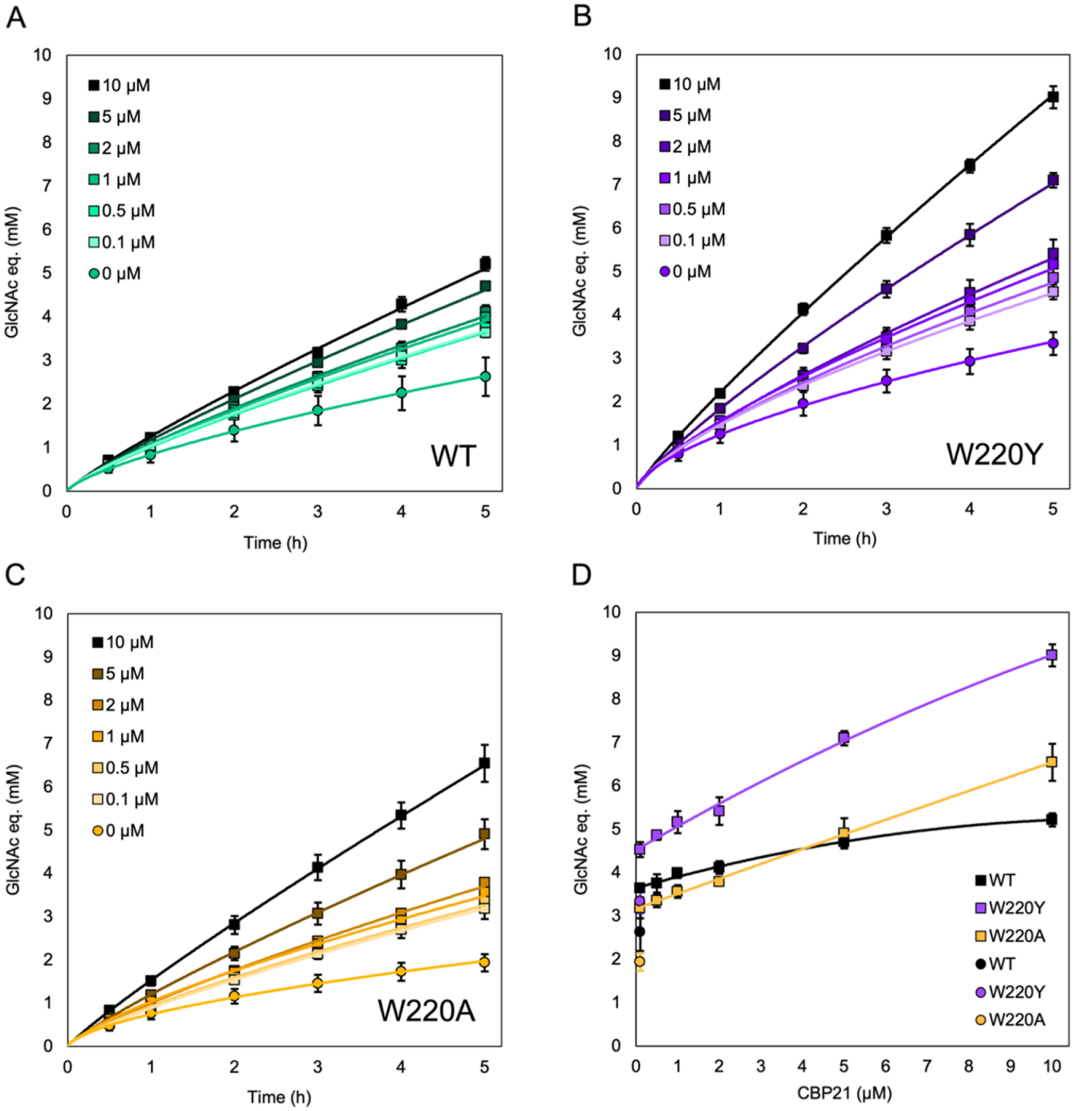
Effect of LPMO concentration on chitin solubilization by *Sm*ChiB, W220Y, and W220A. Chitin solubilization is expressed as the combined amounts of dimers and monomers, in GlcNAc equivalents. (A–C) chitin solubilization by *Sm*ChiB (A) and its W220Y (B) and W220A (C) variants in the presence of varying concentrations of CBP21 indicated in the figure. Next to the control reaction with 0 μM CBP21, a control reaction with 1 μM CBP21 and no ascorbic acid was included (not shown) to assess potential effects of added protein; these two reactions yielded nearly identical product levels. (D) Solubilization after 5 h as a function of the LPMO concentration. The circle symbols on the far left show the solubilization levels from the reactions with 0 μM LPMO. All reactions contained 100 nM Chitinase, 0–10 μM LPMO, and 20 mg/mL β-chitin in 50 mM phosphate buffer, pH 6.0, and were incubated at 37 °C with shaking at 800 rpm. To drive the LPMO reaction, 1 mM ascorbic acid was added. In corresponding control reactions (1 μM LPMO, no reductant), ascorbic acid was replaced with dH_2_O. CBP21 was preincubated with substrate for 30 min, followed by a second 30 min preincubation, in the absence or presence of 1 mM ascorbic acid, before adding the Chitinase to initiate the 5 h time course. Samples were collected hourly starting at 1 h after Chitinase addition. Enzyme-free control reactions, incubated with or without ascorbic acid, did not show any significant product formation (not shown). Error bars represent standard deviations for three independent reactions.

The superior performance of W220Y (relative to W220A) in the presence of CBP21 observed after 6 h (Figure 4) was less pronounced at the 48-h end points (Figure 5). The reactions shown in Figure 5 differed in setup from those in Figure 4, featuring longer incubation times, different LPMOs, and lower reductant concentrations. Nevertheless, the results clearly demonstrate that less processive *Sm*ChiB variants benefit more from LPMO activity than the wild-type enzyme.

### Effects of the LPMO-Dosage

Following the notion that, relative to wild-type *Sm*ChiB, the Trp220 variants profit more from higher substrate concentrations (Figure 2) and the presence of an active LPMO (Figures 4 and 5), higher LPMO activity should benefit them as well. Thus, experiments with varying CBP21 concentrations were carried out. Importantly, in reductant-driven reactions, LPMO activity is limited by the *in situ* generation of H_2_O_2_,^24,26^ which results from both the oxidase activity of the LPMO and abiotic oxidation of the reductant. Thus, there will be a complicated, nonlinear relationship between the LPMO concentration and the actual LPMO activity.^26^

Figure 6 displays a clear dose–response effect and shows that, as expected based on the results discussed above, higher LPMO concentrations are more beneficial for the Trp220 mutants compared to the wild type (Figure 6D)

In line with observations described above, the W220Y mutant outperformed both the wild type and W220A under all conditions, and this performance gap widened with increasing LPMO concentrations. At the highest LPMO concentration, even W220A outperformed the wild-type enzyme (Figure 6D). Figure 6D also shows that just adding a little LPMO has a large effect on chitin solubilization, whereas the addition of larger amounts of LPMO has a more moderate effect that, notably, varies between the *Sm*ChiB variants. For the wild type, the maximum LPMO effect is reached at approximately 1 μM, whereas for the two Trp220 mutants, the effect continues to increase up to the highest LPMO concentration tested. This shows that the wild-type enzyme, with its strong substrate affinity and high processivity, reaches saturating effective substrate concentrations at lower levels of LPMO activity than the weaker-binding Trp220 mutants.

Moreover, increasing the LPMO concentration indeed led to increased levels of oxidized products, which did not vary significantly between the *Sm*ChiB variants (Figure S3). Consistent with the observations discussed above, this shows that the production of solubilized oxidized product was not affected by the nature of the *Sm*ChiB variant.

## Concluding Remarks

Processive glycoside hydrolases are crucial determinants of cellulolytic and chitinolytic enzyme cocktails.^3,50^ Enzyme activity on recalcitrant crystalline substrate regions requires energetically demanding substrate decrystallization to provide enzyme access to an individual polysaccharide chain. By remaining associated with the polymer between successive cuts, processive enzymes face a lower average decrystallization penalty per cleavage than nonprocessive enzymes. This association, and the chitinolytic activity it supports, come at a cost: low off-rates limit turnover when the reaction is not association-limited. In an early study predating the discovery of LPMOs, Horn et al. demonstrated this trade-off for *Sm*ChiB and suggested that enhancing substrate accessibility for nonprocessive enzymes could be more effective than further optimizing processive enzymes themselves.^6^ Leveraging the decrystallizing function of LPMOs is one way to improve substrate accessibility.^27,51^

A first glimpse of this potential strategy for obtaining more efficient chitin degradation was provided by Hamre et al., who showed that some Chitinase variants with reduced processivity and reduced activity on chitin could reach wild type like performance when combined with an LPMO.^31^ The systematic study presented above, focusing on a key determinant of substrate affinity and processivity in *Sm*ChiB, Trp220, sheds further light on this issue and its potential. Using a series of enzymes with varying mutations at position 220, we show that *Sm*ChiB variants with reduced substrate affinity and processivity can perform better than the wild-type enzyme, at higher substrate concentrations and, particularly, if LPMOs are used to increase the effective substrate concentration. With the highest applied LPMO concentration, the W220Y mutant was almost twice as effective as the wild type (Figure 6).

The present study provides fundamental insight into the interplay between chitinases and LPMOs and shows a potential for improving overall chitinolytic efficiency by optimizing both GH affinity and LPMO activity. Efficient chitinolytic enzyme cocktails based on *Serratia* enzymes comprise multiple enzymes, including two processive chitobiohydrolases (*Sm*ChiA and *Sm*ChiB),^52^ and the finetuning of processivity in both these enzymes may be of interest. Further optimization of the LPMO contribution is fully possible, for example by slowly feeding reactions with H_2_O_2_ rather than using the reductant-driven approach.^53^ Such an approach could reduce enzyme consumption if the feeding rate is such that LPMO inactivation is avoided.

Regarding the processivity of *Sm*ChiB, previous studies have shown that Trp220 plays a more critical role than Trp97 and Phe190 within the same aromatic clamp. Our mutational data confirm the importance of a tryptophane residue at position 220, and that aromaticity alone is not sufficient. The processivity data in Figure 3, together with the other experiments reported above, show that the W220Y and W220F mutants differ greatly from the wild-type enzyme, despite the presence of an aromatic residue in position 220.

In conclusion, our results demonstrate intricacies of the interplay between LPMOs and GHs with different processivities and provide novel leads for the design of enzyme systems for solubilizing recalcitrant polysaccharides. When acting alone, processive enzymes rely on their strong substrate affinity to degrade the crystalline substrate, but this dependence is reduced in the presence of an LPMO, which can be exploited to enhance chitin degradation. These novel insights point toward the deployment of new strategies that can be used for efficient enzymatic depolymerization of recalcitrant polysaccharides.

## Supplementary Material















## Notes

Molecular dynamics simulations of *Sm*ChiB variants bound to chitotriose (Figure S1), soluble oxidized products generated during chitin degradation with *Sm*ChiB variants and an LPMO (Figure S2), and formation of oxidized trimers over time at different LPMO concentrations (Figure S3) (PDF)
ChiB-WT (Movie 1) (MPG)
ChiB-W220Y (Movie 2) (MPG)
ChiB-W220F (Movie 3) (MPG)
ChiB-W220H (Movie 4) (MPG)
ChiB-W220Q (Movie 5) (MPG)
ChiB-W220A (Movie 6) (MPG)
