# Supplementary material for: Reduced Processivity in a Chitobiohydrolase Enhances LPMO-Assisted Chitin Depolymerization
Source: Biochemistry. 2026 Feb 17;65(5):591–602. doi: 10.1021/acs.biochem.5c00704 (PMC12961735; doi:10.1021/acs.biochem.5c00704)
Supplement: Supplementary file 1 [file bi5c00704_si_001.pdf]

Supporting Information for

Reduced processivity in a chitobiohydrolase enhances LPMO-assisted chitin depolymerization

Amanda K. Votvik[1], Zarah Forsberg[1], Alfonso Gautieri[2], Vincent G. H. Eijsink[1], Morten Sørlie[1*]

[1] Faculty of Chemistry, Biotechnology, and Food Science, The Norwegian University of Life Sciences (NMBU), 1432 Ås, Norway
[2] Biomolecular Engineering Lab, Dipartimento di Elettronica, Informazione e Bioingegneria, Politecnico di Milano, 20133 Milano, Italy.

[*] To whom correspondence should be addressed: Morten Sørlie (morten.sorlie@nmbu.no)

This PDF file includes:

Figures S1 to S3

In addition, unrestrained trajectories for all ChiB-W220 variants are available as Web Enhanced Objects.

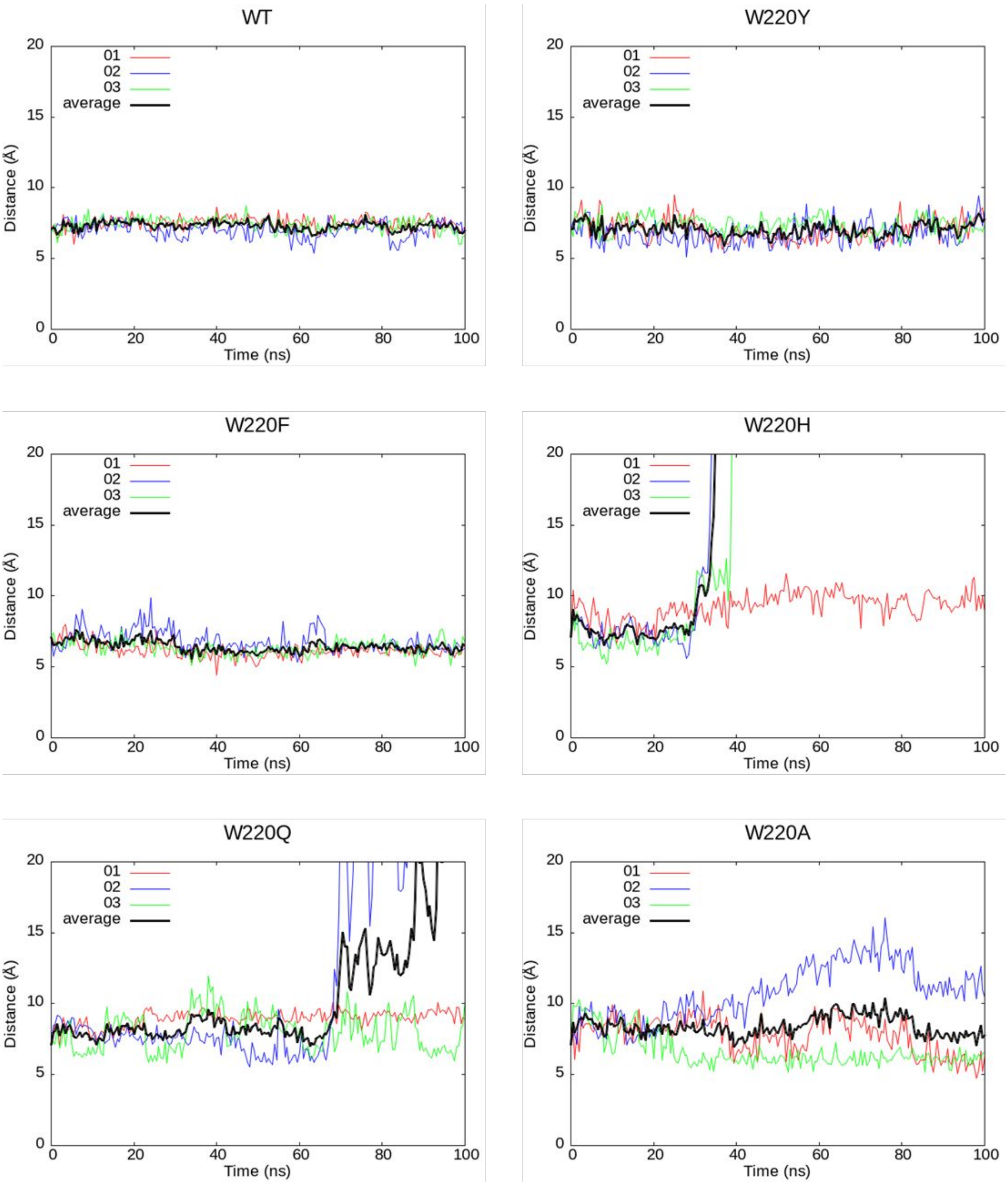

Figure S1. **MD simulations of *Sm*ChiB variants bound to chitotriose.** The graphs show the distance between the Cα of residue 220 and the center of mass of chitotriose for three 100 ns unrestrained MD simulations. The black line shows the average value. Of note, the scale of the Y-axes is set to be from 0 to 20 Å for clarity. In the case of *Sm*ChiB-W220H, the average distance plateaus at ~ 80 Å.

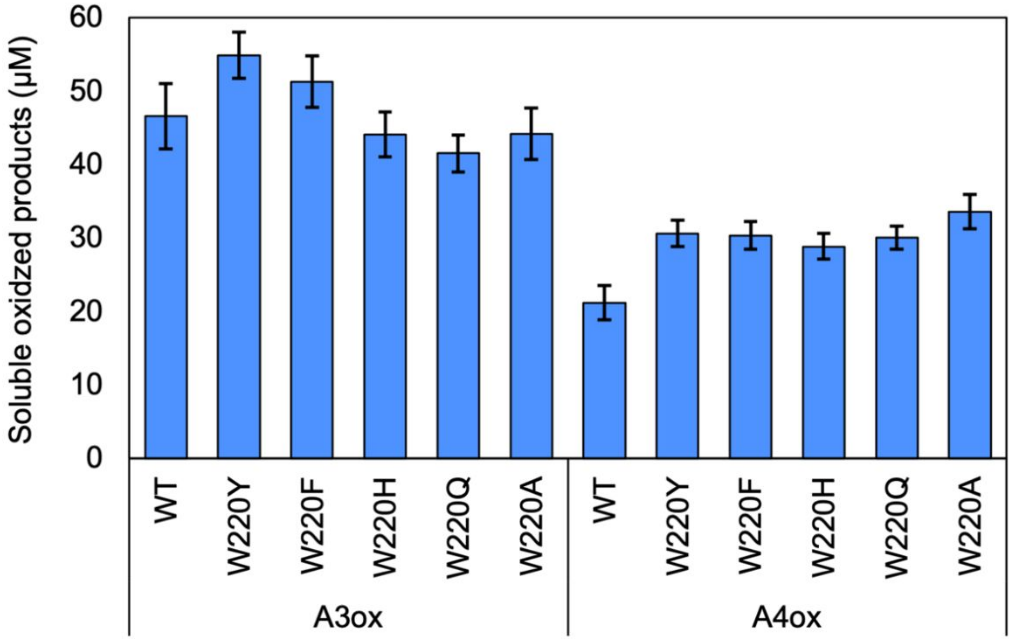

Figure 2S. Soluble oxidized products generated during chitin degradation with *Sm*ChiB variants and an LPMO. The graph shows endpoint levels of oxidized chitotriose (A3ox) and chitotetraose (A4ox) detected in the reactions with active LPMO shown in Fig. 4.

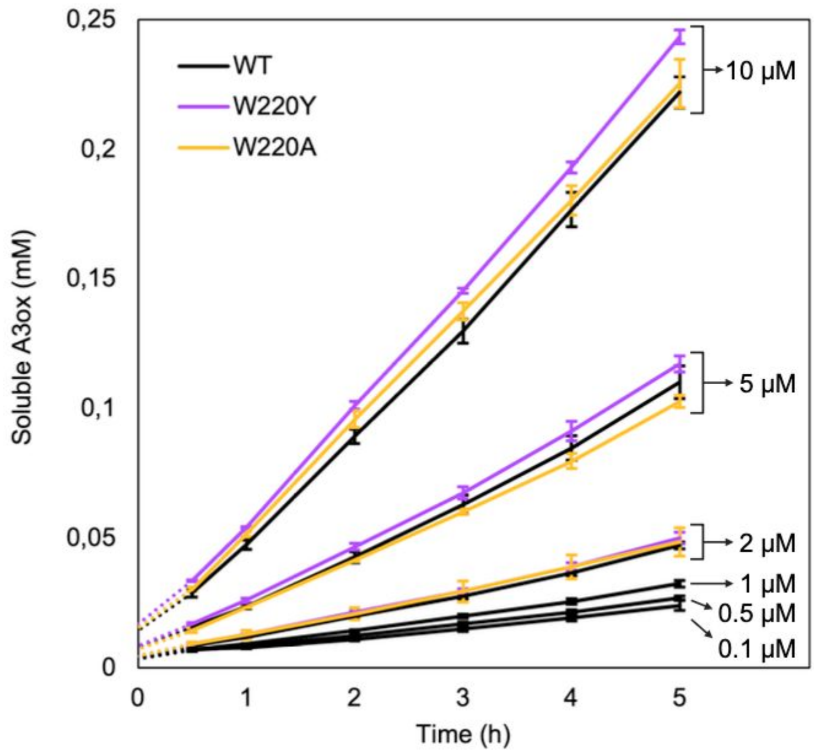

Figure 3S. Formation of oxidized trimers over time at different LPMO concentrations. For LPMO concentrations below 2 μM, only data for the reaction with the wild-type enzyme are shown for clarity. The dotted lines depict the onset of LPMO activation (-0.5 h) and are included only as guides for the eye. Error bars represent standard deviations for three independent reactions.
